# *Listeria monocytogenes* Impact on Mature or Old *Pseudomonas fluorescens* Biofilms During Growth at 4 and 20°C

**DOI:** 10.3389/fmicb.2016.00134

**Published:** 2016-02-15

**Authors:** Carmen H. Puga, Belen Orgaz, Carmen SanJose

**Affiliations:** Department of Nutrition, Food Science and Technology, Faculty of Veterinary, Complutense University of MadridMadrid, Spain

**Keywords:** *Listeria monocytogenes*, *Pseudomonas fluorescens*, biofilms, interspecies interactions, low temperature, CLSM

## Abstract

Changes in spatial organization, as observed by confocal laser scanning microscopy (CLSM), viable cell content, biovolume, and substratum surface coverage of the biofilms formed on glass by *Pseudomonas fluorescens* resulting from co-culture with *Listeria monocytogenes*, were examined. Two strains of *L. monocytogenes*, two culture temperatures and two biofilm developmental stages were investigated. Both *L. monocytogenes* strains, a persistently sampled isolate (collected repeatedly along 3 years from a meat factory) and Scott A, induced shrinkage in matrix volume, both at 20°C and 4°C, in mature or old biofilms, without loss of *P. fluorescens* cell count per surface unit. The nearly homogeneous pattern of surface coverage shown by mono-species *P. fluorescens* biofilms, turned into more irregular layouts in co-culture with *L. monocytogenes*. The upper layer of both mono and dual-species biofilms turned to predominantly consist of matrix, with plenty of viable cells underneath, in old biofilms cultured at 20°C, but not in those grown at 4°C. Between 15 and 56% of the substratum area was covered by biofilm, the extent depending on temperature, time and *L. monocytogenes* strain. Real biofilms in food-related surfaces may thus be very heterogeneous regarding their superficial components, i.e., those more accessible to disinfectants. It is therefore a hygienic challenge to choose an adequate agent to disrupt them.

## Introduction

Known previously as an animal pathogen and ubiquitous in nature, *Listeria monocytogenes* emerged as a foodborne human pathogen in the 1980s (Ryser and Marth, 2007; Warriner and Namvar, 2009). That can be attributed to an unprecedented global improvement of hygienic practices in food industry from the 1970s, including both better cleaning and disinfection methods and a more widespread use of refrigeration. This public health progress, reducing the prevalence of most foodborne diseases, had an undesirable side effect. Elimination by low-temperature of the constraint of microbial competitors implied a new chance for *Listeria*, one of the few psychrotrophic bacterial pathogens, to thrive in refrigerated foods. As adequate storage of pasteurized and/or Ready To Eat (RTE) foods requires low temperatures, cold-tolerant, i.e., psychrotrophic, bacteria tend to be selected in those foods. That is the case of *Pseudomonas* (*fluorescens, putida, fragi*), able to cause important quality defects in protein-rich foods (Andreani et al., 2015).

Coinciding with this trend and the development of microbial ecology approaches to food safety and quality, there has been an increasing interest in biofilms (Costerton et al., 1995; Srey et al., 2013). *Pseudomonas* species were soon characterized as quick and thick biofilm producers, even the non-pathogenic species, often dominant in food spoilage. Their understanding has been driven by the far more abundant clinical and basic information on *Pseudomonas aeruginosa’*s biofilms (Silby et al., 2011; Mann and Wozniak, 2012). Many authors also have studied *L. monocytogenes*’s carrying biofilms (Moretro and Langsrud, 2004; Rieu et al., 2008; Bonsaglia et al., 2014; Guilbaud et al., 2015).

Interactions between *Pseudomonas* and *L. monocytogenes* in biofilms were initially described by Sasahara and Zottola (1993). Their claim on the need of a primary surface colonizer, such as *Pseudomonas* (in that case *P. fragi*) for *L. monocytogenes* attachment, was a very relevant one in its time and not just for the food microbiology field. Multispecies biofilms have attracted attention mostly because their partners can resist harder antimicrobial challenges than single species biofilms (Burmølle et al., 2006; Simões et al., 2009; Sanchez-Vizuete et al., 2015) and because they are now acknowledged to be widely distributed in both natural and industrial environments. Various hypotheses have been used to investigate the specific properties of mixed biofilms and to characterize the interactions between the partners and toward newcomers (Carpentier and Chassaing, 2004; Moons et al., 2009; Yang et al., 2011; Elias and Banin, 2012; Burmølle et al., 2014; Giaouris et al., 2014, 2015; Jahid and Ha, 2014; Bridier et al., 2015) and many attempts have been made to identify the natural biofilm cohabitants at critical sites, including specific food related facilities (Fox et al., 2014; Røder et al., 2015; Rodríguez-López et al., 2015).

New insights on the regulation of biofilm formation are helping to deepen the knowledge about the sort of biofilms that can be found in food industry, where multiple strategies to prevent or delay microbial growth are commonly combined to preserve foods (low temperature, low pH, high osmotic pressure, modified atmospheres, presence of natural antimicrobials, etc.). Food preservation conditions are adverse situations that may activate stress response in some of the present microorganisms, which are thus selected. Certain *Pseudomonas* and *L. monocytogenes* strains belong to those selected at low temperatures (Moretro and Langsrud, 2004; Hemery et al., 2007; Chan and Wiedmann, 2009; Ortiz et al., 2010; Silby et al., 2011; Mann and Wozniak, 2012; Valderrama and Cutter, 2013; Rodríguez-López et al., 2015) and they may jointly form biofilm on raw materials, foods, and inert surfaces at food handling facilities. Though refrigeration tends to be used in food processing and food service facilities during operating hours, higher environmental temperatures tend to occur during pauses or implementation of cleaning and disinfection tasks. Biofilm life may thus switch from 4 to 20°C, or even larger intervals at those sites. *L. monocytogenes* strains that have been found to persist for months or even years (Ortiz et al., 2010; Carpentier and Cerf, 2011) are likely to have often experienced changing culture conditions, apart from partial elimination and repeated sanitizer exposure, by daily but not fully effective cleaning and disinfection cycles. Development of more effective, cheap, and sustainable eradication methods requires more information on the target biofilms where *L. monocytogenes* inhabits.

This study, still in the track of Sasahara and Zottola (1993), tries to follow the formation and aging of *P. fluorescens* and *L. monocytogenes* mixed biofilms in temperature conditions that are realistic for food industry. One *P. fluorescens* and one *L. monocytogenes* strain of food industry origin were used, adding well known *L. monocytogenes* Scott A for comparison. Viability counting was combined with culture-independent evaluations, to get a hint of the heterogeneity in biofilm setups that could be useful for food hygiene purposes. Previous evidence of spatial distribution in these dual-species biofilms has already been reported by the same authors (Puga et al., 2014).

## Materials and Methods

### Bacterial Strains

*Pseudomonas fluorescens* ATCC 948^TM^ and two strains of *L. monocytogenes* were selected as biofilm former organisms. S1 is a *L. monocytogenes* persistent strain, (serotype 1/2a; lineage II) isolated by Ortiz et al. (2010) from an Iberian pig slaughterhouse and its associated processing plant; the other *L. monocytogenes* was the reference clinical strain Scott A (4b; lineage I). All of them were stored at –20°C in Tryptone Soya Broth (TSB, OXOID) with 15% glycerol. Preinocula were obtained in TSB after 24 h incubation at 20°C while shaking (80 rpm) to reach mid exponential phase. Working cultures were obtained from this as follows: 100 μL of preinocula were transferred into a test tube containing fresh TSB and incubated at 20°C for 24 h. Then, cells were harvested by centrifugation at 4000 × *g* for 10 min, washed twice in sterile TSB and their OD_600_ adjusted (0.12), to be used as inocula, in order to reach 10^4^ CFU⋅mL^-1^ for each bacterial strain at the start of either single or binary cultures.

### Experimental System

**FIGURE 1:**
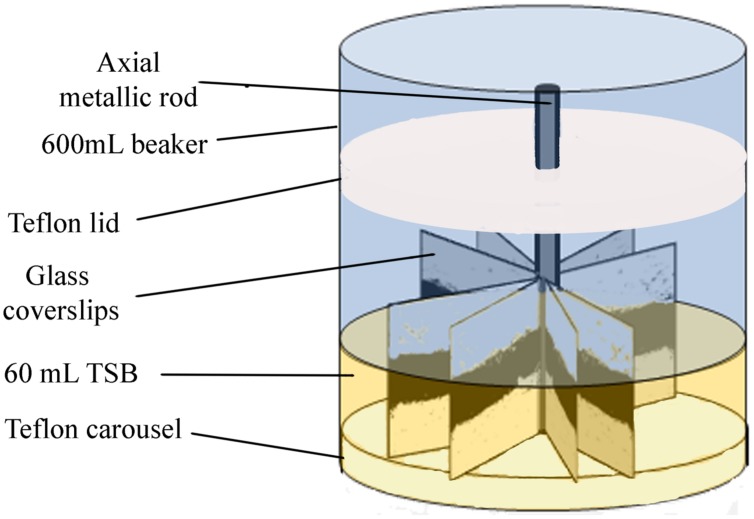
**Scheme of the experimental system used for the development of biofilms**.

Biofilms developed on single-use 22 mm × 22 mm thin, borosilicate commercial microscope glass coverslips. These coverslips provide single-use, relatively wide, clean and undamaged smooth surfaces, without scratches or other microtopographic irregularities, moderately more hydrophilic than stainless steel, and allowing for more reproducible biofilms than reusable metal coupons. As described in Orgaz et al. (2011), 16 coverslips were held vertically by marginal insertion into the narrow radial slits of a Teflon carousel platform (6.6 cm diameter). The platform and its lid were assembled by an axial metallic rod for handling and placed into a 600 mL beaker (**Figure 1**) which was heat-sterilized as a unit, before aseptically introducing 60 mL of inoculated TSB. The glass coupons used in this study as substratum surfaces, were immersed in the liquid culture medium, which covered two thirds of the coupon area. To check whether the covered area was homogeneous in terms of biofilm colonization, the coupon was arbitrarily divided into three equal horizontal bands. The top one, not covered by liquid was the Air-Phase (AP). The intermediate one (ALI), covered and located around the Air-Liquid Interphase, was intensely aerated and exposed to liquid shear during rotation shaking. The Fully Immersed one (FI), less aerated zone, was at the bottom. For multispecies biofilms containing *P. fluorescens* and one of the two *L. monocytogenes* strains afore mentioned, both bacteria were inoculated at the same level (1:1). *P. fluorescens* mono-species biofilms were used as controls. Incubation was carried out at 20°C or 4°C, in a rotating shaker at 80 rpm. Under these conditions, biofilm growth occupied almost 70% of the coverslip’s surface. Samples corresponding to “mature biofilm” were taken after 48 h at 20°C, or 10 days at 4°C. Those taken at 20°C/144 h or 4°C/20 days were here called “old biofilm.”

### Cell Recovery and Counting

For sampling biofilm cells, glass coverslips were withdrawn with tweezers, and were carefully rinsed in sterile 0.9% NaCl to discard weakly attached cells. Then, attached cells of both coverslip faces were removed by swabbing (withdrawing all attached material from both coverslip faces with a cotton swab that was later immersed into an sterile tube containing 1.5 mL peptone water). Tubes were then vigorously stirred in a vortex to break up cell aggregates. Biofilm cells were decimally diluted in peptone water to be plated according to the drop method described by Hoben and Somasegaran (1982). Briefly, three 20 μL drops of each dilution were deposited onto plates of selective media, PALCAM (OXOID) or *Pseudomonas* Agar Base (PAB, OXOID), for counting *Listeria* sp. and *Pseudomonas* sp., respectively, in mono and dual-species biofilms. For purity control, plating on Tryptone Soy Agar (TSA, OXOID) was used to visually detect potential contaminant colonies. Counting was performed after 48 h incubation, at 37°C or 30°C, for *L. monocytogenes* or *P. fluorescens*, respectively. The results presented are the average of two coupons per experiment and three independent experiments (*n* = 6).

### Confocal Laser Scanning Microscopy (CLSM)

The structural effects of *L. monocytogenes* on dual-species biofilms structure were examined by CLSM. For observation, the biofilms developed on the glass coverslips were rinsed with sterile 0.9% NaCl and stained with Syto 13 (S7575, Life Technologies) which labels all bacteria in a population, and CalcoFluor White (18909, FLUKA) a non-specific fluorochrome that binds to cellulose and other polysaccharides present in the extracellular polymeric substances (EPS) biofilm matrix. Thus, for quantification, green here corresponds to cells, whereas blue corresponds to EPS. Five representative regions of 0.12 mm × 0.12 mm located at the air-liquid-interphase zone were selected from each coupon. For this, the side of the coupon (22 mm) was divided into five regions (4.4 mm each one) and the center point of each one was later scanned. CLSM images of these locations were obtained with a FLUOVIEW^®^ FV 1200 Laser Scanning Confocal Microscope (OLYMPUS) and an oil immersion objective lens 60X. Three-dimensional projections (Maximun Intensity Projection, MIP) were reconstructed from z-stacks using IMARIS^®^ 8.1 software (BITPLANE AG, Zurich, Switzerland). The parameter here called *biovolume* was calculated using the MeasurementPro module of IMARIS; the whole image was thus segmented into two channels, green and blue, to estimate the volume occupied by either cells or EPS. The total biovolume (μm^3^) was the sum of cells and EPS biovolumes, using the five fields. Biovolume reduction measurements were here calculated considering the biovolume occupied by *P. fluorescens* in mono-species biofilms represented 100%. The Matrix/Cell ratio was calculated for every image.

### Biomass Determination

**FIGURE 2:**
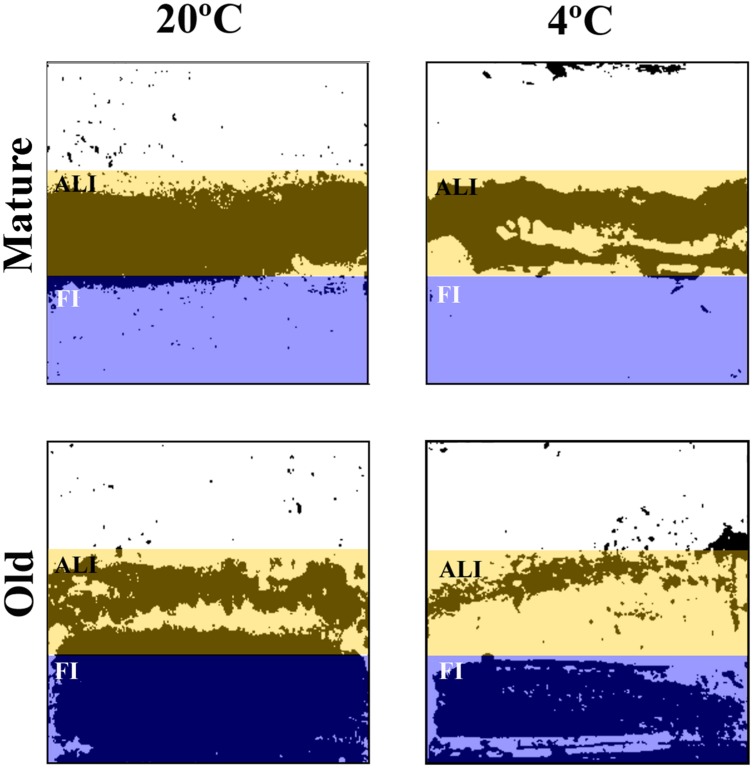
**Scanned coupons black and white images of mature and old *P. fluorescens* mono-species biofilms.** Colored boxes mark the two different areas analyzed. Mature: 20°C/48 h; 4°C/10 days. Old: 20°C/144 h; 4°C/20 days.

To evaluate the surface coverage of the attached biomass (cells plus EPS matrix) five coverslips of each type of biofilms (i.e., young and old biofilms; warm and cold biofilms; *P. fluorescens* mono-species and dual species with *L. monocytogenes*) were dried and stained for 2 min in a 1‰ Coomassie Blue (Brilliant Blue R, SIGMA) solution in acetic acid/methanol/water (1:2.5:6.5) mixture. This step was repeated twice. Once rinsed and dried again, the coverslips were scanned using a 600 dpi resolution (HP Scanjet 300) and analyzed using ImageJ (http://imagej.net). Densitometry allows analyzing the whole area of the stained coupon in comparison with confocal microscopy where fields are much smaller. The aim here was to integrate the biomass results of the whole coupon, segmented in areas with different aeration. A parameter called **% of *covered area*** was estimated for every image. For this, the scanned images were transformed into a binary system (i.e., black and white) and the surface occupied by black was quantified. Each coupon was divided into three zones, as described before. For calculations, the occupation in the air phase was discarded, as that area was scarcely covered, assuming as total biomass coverage the sum of the air-liquid interphase and the fully immersed zone (**Figure 2**).

## Results

### Effects of *L. monocytogenes* Co-culture on *P. fluorescens*’s Biofilm at 20°C

**FIGURE 3:**
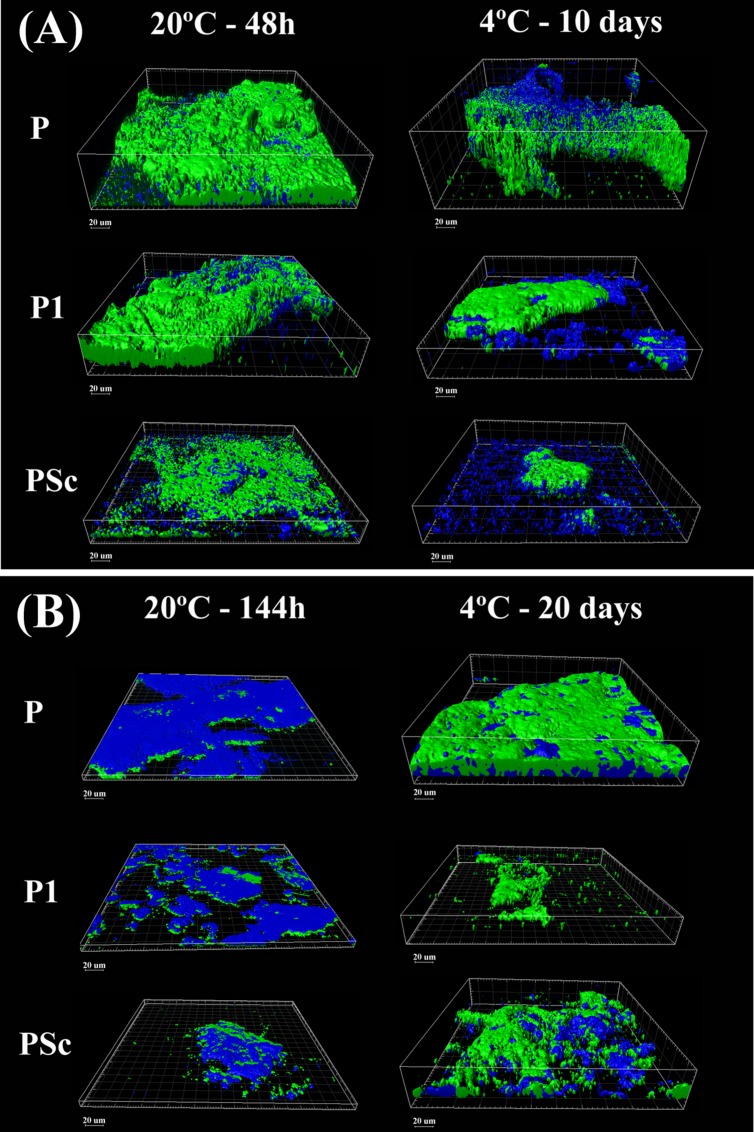
**Three-dimensional CLSM reconstruction of mature **(A)** and old **(B)***P. fluorescens*-carrying biofilms obtained at 20°C or 4°C.** Green: cells; blue: EPS matrix. P: *P. fluorescens* biofilms; P1: mixed *P. fluorescens* and *L. monocytogenes* S1 biofilms; PSc: mixed *P. fluorescens* and *L. monocytogenes* Scott A biofilms.

What is here called “mature” or fully grown biofilm corresponds to the maximum attached population attained in these batch conditions (48 h at 20°C), with around 4 × 10^7^ CFU of *P. fluorescens*/cm^2^ (**Table 1**). At that stage, viable *P. fluorescens* cell numbers experienced almost no change if co-cultured with a *L. monocytogenes* strain. Both strains of *L. monocytogenes* grew more slowly than *P. fluorescens* in the binary biofilms at 20°C, particularly S1 (7.7 log versus 5.9 log). CLSM images, which in this study did not discriminate *P. fluorescens* and *L. monocytogenes* cells (**Figure 3A**), showed a rather homogeneous surface coverage in the case of single species *P. fluorescens* biofilms and a patchy, heterogeneous pattern for the binary biofilms, in spite of the low *L. monocytogenes* numbers (**Table 1**). As seen in **Table 2**, displaying cell and matrix biovolumes, and **Table 3**, presenting biomass distribution and substrate surface occupation, co-culture resulted in a decrease in biofilm biovolume and maximal thickness,. Considering that *P. fluorescens* viable cell number did not decrease, the outcome was a rise in density, in compactness. The matrix to cell ratio (**Table 2**) which was 0.7 in the single species *P. fluorescens* biofilms, was not changed by the presence of the food industry-persistent S1 strain of *L. monocytogenes*, but went down to 0.2 when co-cultured with *L. monocytogenes* Scott A. This strain caused a 75–80% matrix loss in binary biofilms (**Table 2**).

**Table 1:** *P. fluorescens* and *L. monocytogenes* viable cells in biofilms.

Sample^∗^	P	PI		PSc	
	(Log_10_ CFU cm^-2^)	(Log_10_ CFU cm^-2^)		(Log_10_ CPU cm^-2^)	
			
	*P. fluorescens*	*P. fluorescens*	*L. monocytogenes*	*P. fluorescens*	*L. monocytogenes*
	X ± SD	X ± SD	X ± SD	X ± SD	X ± SD
20°C/mature	7.6 ± 0.1^aA^	7.7 ± 0.1^aA^	5.9 ± 0.6^aC^	7.5 ± 0.3^aA,E^	6.7 ± 0.3^aB,C^
20°C/old	5.7 ± 0.1^cB^	6.2 ± 0.1^bA^	4.4 ± 0.2^bc^	6.3 ± 0.0^bA^	5.4 ± 0.1^bB^
4°C/mature	6.6 ± 0.8^b,cA^	6.7 ± 0.1^bA^	3.1 ± 0.1^cC^	6.4 ± 0.0^bA^	4.4 ± 0.2^cB^
4°C/old	6.2 ± 0.1^cA^	5.8 ± 0.7^bA^	5.6 ± 0.2^aA,B^	5.0 ± 0.2^cB,C^	4.6 ± 0.2^cC^


*Different superscripts in small letters mean important statistical differences in columns. Different superscripts in capital letters mean important statistical differences in rows (*n* = 6). ^∗^P: mono-species *P. fluorescens*; P1: *P. fluorescens* and *L. monocytogenes* S1; PSc: *P. fluorescens* and *L. monocytogenes* Scott A. Mature: 20°/48 h and 4°/10 days; Old: 20°/144 h and 4°/20 days.*

**Table 2:** Structural parameters obtained from CLSM images in **Figure 3**.

Sample^∗^	20°C Biofilms			4°C Biofilms		
		
	Max. BF Thickness (μm)	Biovolume reduction (%)	Matrix/ Cells ratio	Max. BF thickness (μm)	Biovolume reduction (%)	Matrix/ Cells ratio
P-mature	43 ± 7		0.7	23 ± 10		0.9
Pl-mature	31 ± 5	27	0.7	29 ± 7	22	0.6
PSc-mature	39 ± 9	55	0.2	13 ± 5	39	0.8
P-old	20 ± 1		1.1	27 ± 13		0.7
Pl-old	21 ± 3	67	1.0	13 ± 4	96	0.1
PSc-old	26 ± 5	73	1.0	26 ± 1	55	0.9


*^∗^P: mono-species *P. fluorescens*; P1: *P. fluorescens* and *L. monocytogenes* S1; PSc: *P. fluorescens* and *L. monocytogenes* Scott A. Mature: 20°/48 h and 4°/10 days; Old: 20°/144 h and 4°/20 days.*

**Table 3:** Structural parameters obtained from scanned coupons analyzed by ImageJ of the biofilms in the ALI: Air-Liquid Interphase and FI: Fully Immersed bands of the coupons shown in **Figure 2**.

Sample^∗^	20°C Biofilms			4°C Biofilms		
		
	Biomass distribution		Covered area (%)	Biomass distribution		Covered area (%)
						
	ALI	FT		ALI	FT	
P-mature	90 ± 3	10 ± 4	30 ± 2^b^	81 –	19 –	26 ± 7^a^
Pl-mature	84 ± 5	15 ± 5	44 ± 5^a^	84 ± 9	16 ± 9	19 ± /^a^
PSc ± mature	99 ± 0	1 ± 0	28 ± /^b^	94 ± 4	6 ± 4	16 ± /^a^
P ± old	35 ± 4	65 ± 4	48 ± 7^b^	21 ± 8	80 ± 8	32 ± 3^a^
Pl ± old	48 ± 4	52 ± 4	57 ± 3^a^	22 ± 6	77 ± 6	36 ± 4^a^
PSc ± old	27 ± 7	73 ± 7	37 ± 4^c^	17 ± 4	83 ± 4	32 ± 3^a^


Different superscripts mean important statistical differences between P, P1 and PSc mature biofilms or old biofilms.*P: mono-species *P. fluorescens*; P1: *P. fluorescens*; and *L. monocytogenes* S1; PSc: *P. fluorescens* and *L. monocytogenes* Scott A. Mature: 20°/48 h and 4°/10 days; Old: 20°/144 h and 4°/20 days.

Binary old biofilms (144 h at 20°C) were clearly into the dispersal stage, having already lost 1–2 log of its viable *P. fluorescens* cells (**Table 1**). By then, *L. monocytogenes* Scott A counts were 1 log less than those of *P. fluorescens* and the S1 strain, 2 log less, though still representing a substantial population in the binary biofilm (2 × 10^4^ CFU cm^-2^; **Table 1**). Maximal biofilm thickness (**Table 2**) in both mono and dual-species biofilms had at that stage decreased by approximately 50% with respect to their corresponding mature biofilms (from 37 to 22 μm on average). It is to be noticed a change in accessibility of the biofilm cells, which appeared then covered by matrix (**Figure 3B**). Remaining cells were thus underneath, packed in a deeper, more protected position, in both the mono and dual-species old biofilms.

### Effects of *L. monocytogenes* Co-culture on *P. fluorescens*’s Biofilm at 4°C

In a previous work of this group (Puga et al., 2014), it was observed that biofilms growing at 4°C for 10 days were approaching the end of the stage featuring a net increase of attached cells per surface unit. Mono-species *P. fluorescens* biofilms matured at low temperature had about 1 log less viable counts/cm^2^ than when matured at 20°C (**Table 1**) and presented about half their maximal thickness (**Table 2**). Just as at 20°C, however, in mature binary biofilms obtained at 4°C, *L. monocytogenes* viable counts were 1–2 log inferior to those of *P. fluorescens*, which remained as in the mono-species controls (**Table 1**). Global biovolume reduction due to co-culture was *L. monocytogenes*-strain dependent, being more severe in old than in mature biofilms (**Table 2**). Regarding matrix distribution, whereas in the mono-species biofilms EPS appeared mostly layered on top of the cells, in the binary biofilms there was a considerable amount of matrix material scattered on void substratum spaces, away from cells (**Figure 3**).

*Pseudomonas fluorescens* population level in the old biofilms (20 days at 4°C) was just slightly lower than in the mature ones (**Table 1**). This could be either due to moderate dispersal or to regrowth, compensating in number the dispersed cells. Global biovolume reduction strongly depended on *L. monocytogenes* strain. It was the food industry-persistent strain S1 that caused more shrinkage in binary biofilms: a 96% loss in biovolume. It also brought about a 50% loss in maximal biofilm thickness and a sharp fall, from 0.6 to 0.1, in matrix to cell ratio (**Table 2**, **Figure 3**).

### Aeration in Mono or Dual-Species *P. fluorescens* Biofilm Development

Every experimental system to develop biofilms has its own particularities. The coupons used in this study have areas with different aeration. To find out how could this influence local biofilm formation and affect the significance of temperature, age, and species interaction, the coupon surface was divided into three band zones as described in the “Material and Methods” section and the biomass attached to each of them (**Figure 2**) was quantified (**Table 3**). As it is shown in the whole coupon images of **Figure 2**, at any incubation moment, important zonal differences in biomass coverage did happen. For one thing, the Fully Immersed surface was colonized after the more aerated zone. Indeed, in mature biofilms most of the biomass was located in the more aerated zone (ALI; ranging from 81 to 99%), whereas in old biofilms the percentage of biomass located in the fully immersed zone (FI) increased (ranging from 52 to 83%; **Table 3**). The latter effect was much more intense in cold biofilms. No significant biomass shrinkage was observed as a result of species interaction, independently of the temperature of biofilms development (**Table 3**). Overall surface coverage reached maxima of 57 and 36% in cultures at 20 and 4°C, respectively. These surface coverages were achieved for the old dual-species biofilms between *P. fluorescens* and *L. monocytogenes* strain S1.

## Discussion

Biofilms formation in the food industry is a serious concern, especially of those where *L. monocytogenes* can persist. More information on these biofilms could be helpful to develop strategies to successfully eradicate them. Nevertheless, conditions usually found in food processing plants, such as low temperature, are often disregarded when developing target biofilms. In this work, the impact that low temperature and biofilm aging have on the population and the structure of mixed biofilms has been evaluated. As biofilm forming microorganisms, one *P. fluorescens* and one *L. monocytogenes* strain of food industry origin were used, plus the reference strain Scott A for comparison. Viability counting was combined with imaging techniques, to gain an insight in the features of these biofilms that could serve as starting point for improving the current cleaning and disinfection strategies.

When surface biomass was measured in the more or less aerated zones of the coupons, it was confirmed that oxygen availability determined a different pattern of surface colonization at the different coupon areas (**Figure 2**). Similar situations can be found in food industry; biofilms with heterogeneous age and physiology are to be expected in close proximity in real locations. Physiological heterogeneity is inherent to complex natural communities (Stewart and Franklin, 2008). On the other hand, these coupons with zonal biofilm heterogeneity, are the ones we use as experimental system. That means that viable cell countings, such as those in **Table 1**, are average measurements, integrating physiologically heterogeneous biofilm situations across a coupon (650 mm^2^ surface) and where at least six coupons were averaged. By comparison, CLSM fields (0.014 mm^2^) show detailed but very localized information (five fields are summed up for volumetric measurements). The two techniques (viable cell counting and CLSM) supply different but complementary information.

The outcome of species interaction on surfaces is assumed to depend on culture conditions, particular species or strains involved, sequence of arrival to the surface (Carpentier and Chassaing, 2004) and the respective population sizes (Mellefont et al., 2008). Regarding population levels, in the present study, those of *L. monocytogenes* were initially as large as those of *P. fluorescens*; this proportion is unrealistic for food industry as a whole, where different species of *Pseudomonas* are far more prevalent. However, high local concentrations of *L. monocytogenes* may occur at particular food industry harborage sites, considering its general endurance (Moretro and Langsrud, 2004; Ryser and Marth, 2007; Valderrama and Cutter, 2013) and a good desiccation survival ability (Alavi and Hansen, 2013). Only two *L. monocytogenes* strains were tested here, but previous studies with different food industry isolates (Puga et al., 2014) support and complement the present results. There, a commensal relationship was found to exist in biofilms between the two species, with a stimulation of *L. monocytogenes* population without an effect on that of *P. fluorescens*, in terms of viable cell numbers. Besides, a stratified species distribution was seen, using specific species labeling, with *L. monocytogenes* occupying the deeper, more anaerobic biofilm layers, in spite of its late incorporation into the biofilm. In the present work, co-culture with *L. monocytogenes* was observed to induce a reduction in *P. fluorescens* biofilm volume (**Figure 3**, **Tables 2** and **3**) without decrease of its cell counts per surface unit (**Table 1**). According to early assumptions on the role of species interaction for joint surface colonization, a good biofilm former species, such as *Pseudomonas*, would play the role of primary colonizer and provide shelter for poor biofilm formers such as *L. monocytogenes* (Sasahara and Zottola, 1993). In this case, *L. monocytogenes* seem to actively redesign the biofilms formed by *P. fluorescens* (Puga et al., 2014) favoring its own proliferation in there and introducing extra compactness in their structure.

*Listeria monocytogenes* counts in this sort of denser biofilms may be underestimated by experimental systems such as those using crystal violet staining, which do not discriminate between cells and matrix. A denser matrix, on the other hand, may contribute to the mechanisms making mixed biofilms more resistant than mono-species ones against external attack with enzymes, antimicrobials, or other agents (Simões et al., 2009; Burmølle et al., 2014; Sanchez-Vizuete et al., 2015). The shrinkage of the matrix could be possibly caused by the production of an additional extracellular matrix component as a result of the interaction between species, such as amyloid fibers. These surface-associated proteins, produced by some members of the Enterobacteriaceae family such as *Escherichia coli* and *Salmonella*, have also been described in certain *P. fluorescens* strains (Larsen et al., 2007; Dueholm et al., 2010; Zhou et al., 2012). In addition, other forms of alteration of the original *P. fluorescens* matrix framework may be involved (Steinberg and Kolodkin-Gal, 2015). According to Periasamy et al. (2015), polysaccharide composition of an individual species significantly impacts mixed species biofilm development and the emergent properties of such communities. If an abundant and extracellular matrix such as that produced by *P. fluorescens* can be considered as “public goods” when shared (Nadell et al., 2009), a reinforced, more compact matrix, induced if not produced, by *L. monocytogenes* in binary biofilms with *Pseudomonas*, could perhaps be considered as *L. monocytogenes*’s contribution to enhanced public goods, providing more protection in spite of less growth, to both partners.

Biofilm aging appeared in this work to involve more changes than cell dispersal, such as structural modifications and cell regrowth. For one thing, not all cells seemed to get detached in these rather old biofilms, just part of them. CLSM images of the old biofilms formed at 20°C (**Figure 3B**) showed that dispersal had cleared out cells from the surface, but many viable cells remained underneath, about 10^6^ *P. fluorescens* CFU cm^-2^ and 10^4^–10^5^ CFU cm^-2^ of *L. monocytogenes* (**Table 1**). There is another aspect worth noting. Practically only the matrix was accessible in those old biofilms. This could at least partly explain the fact that aging adds resistance against stress in general (Lee et al., 2014; Serra and Hengge, 2014). On the other hand, it suggests that enzymatic or other matrix-eroding procedures may be a prerequisite to have access to old biofilm dwelling cells. Another issue related to age is regrowth. Here, no discrimination between residual and fresh cells was made, so it is not possible to know how many of the viable cells in old biofilms are in fact starting a new proliferation cycle.

Biofilm development at 4°C was not merely slower than at 20°C, but cold stress had an impact on biofilm structure. The structural contraction or shrinkage observed as a result of co-culture, was intensified by low temperature and culture time. Besides, biofilms grown at 4°C, particularly binary ones, were more irregular in structure, thickness and matrix distribution (**Figure 3** and **Table 2**). Both *P. fluorescens* and *L. monocytogenes* are known to express at low temperatures a wide range of different membrane components and enzymatic activities (Regeard et al., 2000; Hemery et al., 2007; Chan and Wiedmann, 2009; Durack et al., 2013); some of them could be involved in the development of the mentioned biofilm features.

## Conclusion

When this dual-species consortium develop biofilms on a solid surface, apparently species interaction, cold stress and aging contribute to a more compact structure than the one built by *P. fluorescens* in single species biofilms at 20°C. The actual change in the matrix framework and the mechanism to obtain it, deserves further work, as the pathogen’s shelter is thus reinforced. The types of biofilms resulting from the interaction between *P. fluorescens* and *L. monocytogenes*, cold stress and aging could be used as targets for cleaning and disinfection procedures.

## Author Contributions

CP: conception of the work; analysis and interpretation of the data; drafting the work. BO: design of the work; interpretation of the data; drafting and revising the work; Agreement to be accountable for all aspects of the work in ensuring that questions related to the accuracy or integrity of any part of the work are appropriately investigated and resolved. CS: drafting the work and revising it critically; Agreement to be accountable for all aspects of the work in ensuring that questions related to the accuracy or integrity of any part of the work are appropriately investigated and resolved.

## Conflict of Interest Statement

The authors declare that the research was conducted in the absence of any commercial or financial relationships that could be construed as a potential conflict of interest.
